# Supplementary material for: Transcriptome Analysis Reveals Vimentin-Induced Disruption of Cell–Cell Associations Augments Breast Cancer Cell Migration
Source: Cells. 2022 Dec 13;11(24):4035. doi: 10.3390/cells11244035 (PMC9776984; doi:10.3390/cells11244035)
Supplement: Supplementary file 1 [file cells-11-04035-s001.zip › DEGs and enrichment analysis.zip!/DEGs and enrichment analysis/WT_VIMvsVector KEGG/ALL/WT_VIMvsVector_ALL.pathway_map.zip!/WT_VIMvsVector_ALL.pathway_map/src/hsa05130.html]

hsa05130


- hsa:10376

- Up regulated genes

ENSG00000123416(2.28022518189755)

- hsa:203068

- Up regulated genes

ENSG00000214222(3.02126849482465)
- hsa:10382

- Up regulated genes

ENSG00000104833(2.74968979348514)

Close
